# Prevalence and determinants of early initiation of breastfeeding (EIBF) and prelacteal feeding in Northern Ghana: A cross-sectional survey

**DOI:** 10.1371/journal.pone.0260347

**Published:** 2021-11-22

**Authors:** Stephen Dajaan Dubik, Kingsley E. Amegah

**Affiliations:** 1 Department of Nutritional Sciences, University for Development Studies, Tamale, Ghana; 2 Department of Public Health, Catholic University College of Ghana, Fiapre, Ghana; 3 Department of Health Information, Hohoe Municipal Hospital, Ghana Health Service, Hohoe, Ghana; Universita degli Studi dell’Insubria, ITALY

## Abstract

**Background:**

There is suboptimal early initiation of breastfeeding (EIBF) with widespread prelacteal feeding in Ghana. However, studies exploring the determinants of EIBF and prelacteal feeding are limited in Ghana. The study was conducted to assess the prevalence and determinants of EIBF and prelacteal feeding in Northern Ghana.

**Methods:**

This cross-sectional study was conducted among 508 mothers with infants aged 0–24 months in the Sagnarigu Municipality of Northern Ghana. The quantitative data were collected using a structured questionnaire adapted from Ghana’s demographic and health survey. Multivariate logistic regression was used to identify the independent determinants of EIBF and prelacteal feeding.

**Results:**

The prevalence of EIBF and prelacteal feeding was 72% and 21%, respectively. The independent positive determinants of EIBF were partner support to breastfeed [adjusted Odds ratio (AOR): 1.86, 95% Confidence interval (CI): 1.09–3.17] and exposure to breastfeeding information during pregnancy (AOR = 1.63 (95% CI: 1.01–2.64). Lower odds of EIBF were observed among mothers from extended family (AOR = 0.62, 95% CI: 0.41–0.95). Regarding prelacteal feeding, negative determinants were having a normal weight baby (AOR: 0.50, 95% CI: 0.27–0.90), exposure to breastfeeding information during pregnancy (AOR: 0.54, 95% CI: 0.31–0.92), while experiencing delayed onset of lactation was a risk factor for prelacteal feeding practice (AOR: 2.35, 95% CI: 1.41–3.94).

**Conclusion:**

In this study, EIBF was slightly higher than the 2030 global target on EIBF with widespread prelacteal feeding practice. Health programs aimed at improving EIBF should focus on the women partners, nutrition counselling, and support to mothers from the extended family. In the same vein, programs aimed at discouraging prelacteal feeding practice should also target women at risk, such as those with low birthweight babies and women experiencing delayed lactation onset.

## Introduction

Early breastfeeding initiation is defined as starting human breastmilk feeding within one hour after birth [1]. It plays a crucial role in reducing the risk of morbidity and mortality among neonates and infants. For neonates, EIBF plays an essential role in reducing early new-born danger signs and severe illness, thereby reducing the risk of mortality in new-borns [2, 3]. Studies also show EIBF minimizes the risk of infant morbidity such as diarrhoea, prevents undernutrition, and helps infants fight infections. Therefore, EIBF is principal in increasing the survival rates of children [2, 4, 5]. The forgoing evidence of the benefits of EIBF informed the recommendation by World Health Organisation (WHO) that breastfeeding should be initiated within one hour after delivery [6]. Studies also indicate that delayed breastfeeding initiation increases the risk of neonatal morbidity and mortality [7, 8].

One of the practices that hamper EIBF and other optimal breastfeeding practices is prelacteal feeding. Prelacteal feeding is linked to poor child health outcomes. Data from studies show the practice of prelacteal feeding increases the risk of infections and hospitalisations in infants [9] and limits an infant’s frequency of suckling [10]. Despite these health consequences, prelacteal feeding is widely practiced in many countries [11, 12]. For example, in Sub-Saharan African countries, prelacteal feeding practice is prevalent (32%) [12].

Despite the enormous benefits of EIBF stated above, the prevalence of EIBF is suboptimal worldwide [13]. For instance, the global breastfeeding scorecard data indicates that only 43% of new-borns were put to the breast within one hour of birth in 2019. The situation is worst in African countries, where the prevalence of EIBF ranges from 38% in Central African to 69% in Southern Africa [12]. In Ghana, recent data indicates a decline in EIBF from 56% in 2014 to 52% in 2017, with a relatively high prevalence of prelacteal feeding (15%) [10, 14]. This situation in Ghana is worrisome since neonatal/infant morbidity, mortality, and undernutrition are widespread [10, 14].

Individual, community, and health system factors predict early breastfeeding initiation [15–17] and prelacteal feeding [18–20]. In Ghana, studies examining the determinants of EIBF are limited [15], with a dearth of evidence of prelacteal feeding determinants. Also, since breastfeeding initiation and prelacteal feeding practices are often influenced by culture, health-related, and sociodemographic factors, exploring the determinants of EIBF and prelacteal feeding in this study population may form the basis for future interventions to improve breastfeeding practices in the study area. Therefore, this study aimed to investigate the prevalence and determinants of EIBF and prelacteal feeding in Northern Ghana.

## Materials and methods

We conducted a cross-sectional study among 508 women with children 0–24 months in Sagnarigu Municipality of Northern Ghana. Sagnarigu Municipality has 79 communities, with three Quasi-Government Hospitals, six privately-owned hospitals, and many primary health facilities. The Sagnarigu Municipality has a total population of 148,099, with males constituting the majority of the population. The municipality covers a land size of 200.4km2 and shares boundaries withTamale metropolis, Savelugu-Nanton, Tolon, and Kumbungu [16].

We included mothers with children 0–24 months. For a mother to be included in the study, she had to be a permanent resident in the municipality and have a child between 0–24 months. Mothers who were sick and those with children older than 24 months were excluded.

We calculated the sample size using a single proportion formula with the following parameters. Critical value = 1.96 at 95% CI, level of precision = 0.05, design effect = 1.5, and prevalence of EBF from a previous study = 27% [17]. The study was part of a larger study to assess the determinants of optimal breastfeeding, breastfeeding challenges, and coping strategies. The prevalence of EBF was used in calculating the sample size since data on EBF prevalence was available from a study conducted in the Tamale metropolis. Our study setting, Sagnarigu Municipality, was carved out of the Tamale metropolis. Therefore, the Tamale metropolis and Sagnarigu share similar characteristics.

Sampling was done through multi-stage sampling techniques. In the first stage, Sagnarigu Municipality was divided into rural and urban communities, and four communities, each from rural and urban, were conveniently selected to participate in the study. The sample size of 508 was proportionally allocated to the rural and urban communities based on the estimated population of women of reproductive age. The study participants (mothers with children 0–24 months) were selected through consecutive sampling. With consecutive sampling, a mother who came for child welfare clinic and met the inclusion criteria was invited to participate in the study until the desired sample was reached. The period for study participants recruitment ranges from March 2020 to May 2020.

Data was collected using a previously validated questionnaire in a similar population. The questionnaire for the study was adopted from Ghana’s Demographic and health survey (GDHS) [10]. One-day training workshop was organised for the data collectors. We trained the data collectors on administering the questionnaire, ethics related to data collection, and the study’s objectives. The questionnaire consisted of section A: sociodemographic characteristics, section B: household wealth index, section C: breastfeeding and antenatal care attendance and section D: mothers’ knowledge of breastfeeding.

The study’s explanatory variables include age, marital status, religion, level of education, ethnicity, type of family, mother working, occupation, number of children, place of residence, age of infant in months, child sex, and birth weight. Other covariates include trimester of first Antenatal care (ANC) attendance, number of ANC attendance [<8 (inadequate), ≥8 (adequate)], received breastfeeding information, place of delivery, mode of delivery, birth attendant, delayed onset of lactation, previous breastfeeding experience, partner support and breastfeeding knowledge level. **Delayed onset of lactation** was evaluated by asking the following question “After birth, how many days did it take for your breastmilk to start flowing?”, ≤3 days were coded “no” for delayed onset of lactation, and >3 days were coded “yes” for delayed onset of lactation. **Knowledge of breastfeeding** was scored on 14 items. This was related to the timing of breastfeeding initiation, colostrum feeding, the value of breastmilk, exclusive breastfeeding, and other breastfeeding recommendations. A correct response was scored 1, while an incorrect answer was scored 0, with a total maximum score of 14. The total score of each pregnant woman was summed and converted to 100 percent for interpretation. A percentage score of ≤ 70% was considered a low breastfeeding knowledge level, while a percentage score of > 70% was considered a high breastfeeding knowledge level. **The household wealth index** was determined according to the GDHS approach using principal component analysis [10].

The outcome variables were EIBF and prelacteal feeding. Early initiation of breastfeeding was defined as initiating breastfeeding within one hour after birth. Mothers who reported initiating breastfeeding within one hour of birth were coded “Yes” for EIBF, while mothers who reported initiating breastfeeding after 1 hour of birth were coded “No” for EIBF. Prelacteal feeding was defined as providing foods and/or drinks other than human milk within the first three days after delivery. Mothers who reported giving other foods and/or drinks within three days after delivery were coded “Yes” for prelacteal feeding, while mothers who reported not providing any food and/or drink to the infant within three days after delivery were coded “No” for prelacteal feeding.

Data were analysed using STATA 16.0. We used the Chi-square test to distribute study participants characteristics according to EIBF status and prelacteal feeding. The multivariate logistic regression analysis included variables (p<0.25) after the chi-square test. The multivariate logistic regression was performed using forward-stepwise variable selection criteria to include variables that were significant at p<0.25 during the chi-square test. Two models were fitted in the logistic regression. Model 1 examined the association between the predictor variables and early breastfeeding initiation, while Model 2 examined the association between the predictor variables and prelacteal feeding. Confidence interval at 95% confidence interval and odds ratios were used to report each variable’s strength of association at a significance level of P-value less than 0.05.

### Ethics statement

The study received ethical approval from the Committee on Human Research and Publication Ethics of the School of Medical Sciences, Kwame Nkrumah University of Science and Technology (Ref: CHRPE/AP/044/20). Written informed consent was obtained from all study participants.

## Results

### Distribution of study participants characteristics by early initiation of breastfeeding and prelacteal feeding

Table 1 presents the characteristics of the study participants by EIBF and prelacteal feeding. The proportion of EIBF was 364/508 (72%). There was a significant association between type of family, number of antenatal care (ANC) attendance, breastfeeding information during pregnancy, mode of delivery, perceived partner support, and EIBF. The rest of the covariates did not show significant associations (Table 1).

**Table 1:** Distribution of respondent’s characteristics by early initiation of breastfeeding and prelacteal feeding.

Variables	Early Initiation of breastfeeding			Prelacteal feeding		
	No (%)	Yes (%)	*P-value*	No (%)	Yes (%)	*P-value*
Mothers age (In years)						
<30	90 (17.7)	231 (45.5)	0.840	249 (49.0)	72 (14.2)	0.152
≥30	54 (10.6)	133 (26.2)		155 (30.5)	32 (6.3)	
Marital status						
Married	141 (27.8)	361 (71.1)	0.236	400 (78.7)	102 (20.1)	0.432
Single	3 (0.6)	3 (0.6)		4 (0.8)	2 (0.4)	
Religion						
Islam	130 (25.6)	335 (65.9)	0.522	368 (72.4)	97 (19.1)	0.476
Christianity	14 (2.8)	29 (5.7)		36 (7.1)	7 (1.4)	
Ethnicity						
Dagomba	121 (23.8)	303 (59.6)	0.830	337 (66.3)	87 (17.1)	0.954
Other ethnicity	23 (4.5)	61 (12.0)		67 (13.2)	17 (3.3)	
Level of education						
No education	79 (15.6)	190 (37.4)	0.574	210 (41.3)	59 (11.6)	0.598
Primary	26 (5.1)	82 (16.1)		87 (17.1)	21 (4.1)	
Secondary	26 (5.1)	54 (10.6)		63 (12.4)	17 (3.3)	
Tertiary	13 (2.6)	38 (7.5)		44 (8.7)	7 (1.4)	
Type of family						
Nuclear family	39 (7.7)	137 (27.0)	**0.024**	146 (28.7)	30 (5.9)	0.163
Extended family	105 (20.7)	227 (44.7)		258 (50.8)	74 (14.6)	
Currently working						
No	34 (6.7)	63 (12.4)	0.103	77 (15.2)	20 (3.9)	0.968
Yes	110 (21.7)	301 (59.3)		327 (64.4)	84 (16.5)	
Occupation						
Agric/farming	8 (1.9)	17 (4.1)	0.609	17 (4.1)	8 (1.9)	0.467
Public/civil servant	9 (2.2)	35 (8.5)		36 (8.8)	8 (1.9)	
Trader	59 (14.4)	147 (35.8)		167 (40.6)	39 (9.5)	
Hairdresser/dressmaker	34 (8.3)	102 (24.8)		107 (26.0)	29 (7.1)	
Wealth Quintile						
Poorest	34 (6.7)	72 (14.3)	0.113	80 (15.8)	26 (5.1)	0.213
Second	34 (6.7)	62 (12.3)		80 (15.8)	16 (3.2)	
Middle	19 (3.8)	82 (16.2)		86 (16.6)	18 (3.4)	
Forth	29 (5.7)	72 (14.3)		74 (14.7)	27 (5.3)	
Richard	27 (5.3)	74 (14.7)		84 (16.6)	17 (3.4)	
Number of children						
<3	78 (15.4)	177 (34.8)	0.260	196 (38.6)	59 (11.6)	0.135
≥3	66 (13.0)	187 (36.8)		208 (40.9)	45 (8.9)	
Place of residence						
Rural	61 (12.0)	123 (24.2)	0.070	148 (29.1)	36 (7.1)	0.703
Urban	83 (16.3)	241 (47.4)		256 (50.4)	68 (13.4)	
Age of child (In Months)						
< 10 months	112 (22)	286 (56.3)	0.845	312 (61.4)	86 (16.9)	0.228
≥10 months	32 (6.3)	78 (15.4)		92 (18.1)	18 (3.5)	
Sex of child						
Male	68 (13.4)	175 (34.4)	0.862	188 (37.0)	55 (10.8)	0.248
Female	76 (15.0)	189 (37.2)		216 (42.5)	49 (9.6)	
Normal birthweight						
No	23 (4.5)	40 (7.9)	0.125	40 (7.9)	23 (4.5)	**0.001**
Yes	121 (23.8)	324 (63.8)		364 (71.7)	81 (15.9)	
Trimester of first ANC attendance						
First trimester	60 (11.8)	191 (37.6)	0.084	210 (41.3)	41 (8.1)	0.062
Second trimester	82 (16.1)	168 (33.1)		188 (37.0)	62 (12.2)	
Third trimester	2 (0.4)	4 (0.8)		5 (1.0)	1 (0.2)	
Number of ANC attendance						
Inadequate	102 (20.1)	216 (42.5)	**0.016**	247 (48.6)	71 (14)	0.180
Adequate	42 (8.3)	148 (29.1)		157 (30.9)	33 (6.5)	
Breastfeeding information during pregnancy						
No	42 (8.3)	61 (12.0)	**0.002**	67 (13.2)	36 (7.1)	**<0.001**
Yes	102 (20.1)	303 (59.6)		337 (66.3)	68 (13.4)	
Place of delivery						
Health facility	128 (25.2)	317 (62.4)	0.579	352 (69.3)	93 (18.3)	0.527
Home	16 (3.1)	47 (9.3)		52 (10.2)	11 (2.2)	
Mode delivery						
Vaginal	121 (23.8)	333 (65.6)	**0.014**	369 (72.6)	85 (16.7)	**0.005**
Cesarean	23 (4.5)	31 (6.1)		35 (6.9)	19 (3.7)	
Birth Attendant						
Skilled	126 (24.8)	318 (62.6)	0.966	352 (69.3)	92 (18.1)	0.715
Unskilled	18 (3.5)	46 (9.1)		52 (10.2)	12 (2.4)	
Delayed onset of lactation						
No	110 (21.7)	303 (59.6)	0.074	344 (67.7)	69 (13.6)	**<0.001**
Yes	34 (6.7)	61 (12.0)		60 (11.8)	35 (6.9)	
Previous breastfeeding experience						
No	40 (7.9)	74 (14.6)	0.070	86 (16.9)	28 (5.5)	
Yes	104 (20.5)	290 (57.1)		318 (62.6)	76 (15.0)	
Perceived Partner support to breastfeed						
No	37 (7.3)	45 (8.9)	**<0.001**	54 (10.6)	28 (5.5)	**0.001**
Yes	107 (21.1)	319 (62.8)		350 (68.9)	76 (15.0)	
Knowledge of Breastfeeding						
Low	8 (1.6)	30 (5.9)	0.300	27 (5.3)	11 (2.2)	0.178
High	136 (26.8)	334 (65.7)		377 (74.2)	93 (18.3)	

The proportion of prelacteal feeding practice among the mothers was 104/508 (20.5%). There were significant differences between birth weight, breastfeeding information, mode of delivery, delayed onset of lactation, perceived partner support to breastfeed, and prelacteal feeding practice. There were no significant differences between the rest of the variables and prelacteal feeding practice (Table 1).

### Determinants of EIBF and prelacteal feeding practice among mothers in Sagnarigu Municipality

The multivariate analysis revealed type of family, exposure to breastfeeding information, and partner support to breastfeed as independent determinants of EIBF. Lower odds of EIBF were observed among mothers from the extended family (AOR = 0.62 (95% CI: 0.41–0.95). On the contrary, higher odds of EIBF were observed among mothers who were exposed to breastfeeding information (AOR = 1.63 (95% CI: 1.01–2.64) and those who reported receiving support to breastfeed from their partners (AOR = 2.09 (95% CI: 1.24–3.50).

From model 2 in Table 2, multivariate logistic regression analysis revealed birthweight, exposure to breastfeeding information, and delayed lactation onset as predictors of prelacteal feeding practice. Prelacteal feeding practice was less likely among mothers with normal weight babies [AOR = 0.48 (95% CI: 0.27–0.87)], and mothers who received breastfeeding information during pregnancy [AOR = 0.46 (95% CI: 0.28–4.17)]. Risk factor for prelacteal feeding practice was experiencing delayed onset of lactation [AOR = 2.52 (95% CI: 1.52–4.17)].

**Table 2:** Determinants of early breastfeeding initiation and prelacteal feeding among mothers in Sagnarigu Municipality.

MODELS	Model 1		Model 2	
Predictor Variables	Early Initiation of breastfeeding		Prelacteal feeding	
	AOR (95%CI)	P-value	AOR (95%CI)	P-value
Type of family				
Nuclear family	1.00			
Extended family	0.62 (0.41–0.96)	**0.032**		
Normal birthweight				
No			1.00	
Yes			0.48 (0.27–0.87)	**0.016**
Breastfeeding information during pregnancy				
No	1.00		1.00	
Yes	1.63 (1.01–2.64)	**0.047**	0.46 (0.28–4.17)	**0.003**
Delayed onset of lactation				
No			1.00	
Yes			2.52 (1.52–4.17)	**< 0.001**
Partner support to breastfeed				
No	1.00			
Yes	2.09 (1.24–3.50)	**0.005**		

Adjusted Odds Ratio; Model 1 examined the relationship between predictor variables and early Initiation of breastfeeding, Model 2 examined the relationship between predictor variables and Prelacteal feeding.

## Discussion

The prevalence of EIBF was 72%, higher than that of the 2014 GDHS (56%) [10] and 2017/2018 Ghana multi indicator Survey (MICS) (52%) [14]. The differences may be due to the sampling techniques. The study participants were selected using non-probability sampling techniques, while GDHS and MICS utilize probability sampling techniques. Furthermore, in this study, a significant majority of the study participants were from urban communities. Women from the urban areas have unimpeded access to various levels of medical facilities. Access to medical facilities is indicative of EIBF [18, 19]. This may be the possible explanation for the high prevalence of EIBF reported in this study.

According to WHO, the EIBF rating of 72% should be described as good [20]. Generally, the prevalence of EIBF was, therefore, good in the Sagnarigu Municipality of Northern Ghana. The prevalence of EIBF reported in this study is higher than other studies in Tanzania (51%) [18] and Nigeria (34.7%) [21], but lower than what has been reported in Ethiopia (75%) [22] and a prospective study in low and middle-income countries (75%) [23]. The possible explanation for the differences in rates of EIBF may be due to sociodemographic characteristics of the study subjects, culture, religion, and variation in methodology. Even though the prevalence of EIBF in this study was good, it falls short of WHO recommendation that all newly-born infants should be put to the breast within one hour after birth [6].

Partner support to breastfeed was found as an independent determinant of EIBF. A previous study has reported the role of partner support in women’s breastfeeding success [24]. Perhaps, the support received by women from their partners motivates them to initiate breastfeeding early. In this dominant Muslim population, breastfeeding interventions and programmes should consider partners’ involvement in improving EIBF rates. However, it is essential to emphasize the role of maternity staff in enhancing the rates of EIBF. For instance, a study in South Sudan reported the significant role of healthcare workers in improving the prevalence of EIBF [25].

We observed higher odds of EIBF among mothers who were exposed to breastfeeding information during pregnancy. This finding has been reinforced in previously published studies [26, 27]. On the contrary, a systematic review suggests no conclusive evidence on the effect of breastfeeding education and EIBF [28]. These discrepancies may be due to different contexts and circumstances of those receiving the counselling.

We found lower odds of EIBF among mothers from extended family. A similar study conducted in Ethiopia also concluded that mothers who lived with extended family are less likely to initiate breastfeeding early [29]. This may be due to the presence of respected people in the extended family, such as grandmothers, who sometimes influence the introduction of ritual fluids, thereby delaying the EIBF. Indeed, the power of grandmothers in breastfeeding initiation has been reinforced in rural Northern Ghana [30]

Other covariates such as birth weight, mode of delivery, and delayed onset of lactation did not emerge as determinants of EIBF. This is contrary to previous studies where birth weight [23] and mode of delivery [23] were associated with EIBF.

The prevalence of prelacteal feeding was 21%, meaning prelacteal feeding was widespread among the women. The prevalence of prelacteal feeding places our study below studies conducted in Pakistan (65%) [31] and South Sudan (53%) [32]. However, the prevalence of prelacteal feeding in this study is higher than what has been reported in previous studies in Ethiopia [33] and among mothers with low birthweight babies in Ghana [34]. These differences may be attributed to variations in the studies methods and cultural beliefs about prelacteal feeding. Indeed, culture has been cited as the basis for providing prelacteal feeds in Eastern Ethiopia [33]. Furthermore, evidence from Nigeria shows both medical and non-medical staff routinely give prelacteal feeds to new-borns [35]. While medical staff gives mainly for perceived milk insufficiency, prevention of dehydration, hypoglycaemia, and neonatal jaundice, non-medical staff provides prelacteal feeds to prepare the child’s gastrointestinal tract for digestion and to quench thirst [35]. To add, Asim and colleagues in Pakistan assert that healthcare workers at private health facilities sometimes introduced prelacteal feeds to neonates without parents’ consent [31]. This may be the reason for the high prevalence of prelacteal feeding in their study compared to the present research. There is a need for health promotion programs and community engagement on the harmful effect of prelacteal feeding in our study area. Additionally, training programs for medical and non-medical staff is also crucial in reducing the prevalence of prelacteal feeding.

We found birth weight as one of the critical determinants of prelacteal feeding. Mothers with normal birth weight babies had lower odds of giving prelacteal feeds to their infants. In a similar study in Western Nepal, low birth weight babies had higher odds of receiving prelacteal feeds [36]. Mothers with low birth weight babies encounter many problems such as delayed first suckling and poor or no suckling, which may motivate mothers to give prelacteal foods [37]. This may be the possible explanation for higher prelacteal feeding among low birthweight babies and contrary among normal birth weight babies in this study.

Receiving breastfeeding information during pregnancy was also a predictor of prelacteal feeding. Mothers who received breastfeeding information had lower odds of giving prelacteal feeds to their infants. This agrees with a previous study in South Sudan, where mothers who received breastfeeding counselling had lower odds of introducing prelacteal feeds to their infants [38]. In Ethiopia, lack of counselling about breastfeeding was associated with an increased likelihood of giving prelacteal foods [33]. Breastfeeding counselling is critical in discouraging the practice of prelacteal feeding in developing countries.

Our findings suggest that prelacteal feeding was widely practiced by mothers experiencing a perceived delayed onset of lactation after birth. In Nigeria and Ethiopia, mothers cited initial delay in breastmilk flow [39] and insufficiency of breastmilk [33] as the basis for giving prelacteal foods. Breastfeeding support and counselling should target women experiencing delayed onset of lactation. This is crucial in minimising prelacteal feeding risk among women experiencing the delayed onset of lactation after birth.

The study has limitations. First, study communities and participants were selected using non-probability sampling techniques. This could lead to sampling bias, thereby affecting how the study findings can be generalized to the entire study area. Secondly, breastfeeding initiation and prelacteal feeding practice were self-reported by the women. This could lead to social desirability bias, thereby influencing the prevalence of EIBF and prelacteal feeding. Lastly, breastfeeding initiation and prelacteal feeding were recalled by the women. This could also lead to recall bias. Furthermore, the study could not assess the maternity practices of the study participants, which could also influence EIBF and prelacteal feeding.

Notwithstanding these limitations, to the best of our knowledge, this is the first study to specifically investigate the determinants of EIBF and prelacteal feeding in the Sagnarigu Municipality of Ghana.

## Conclusions

The prevalence of EIBF was good, slightly higher than the 2030 global target on EIBF with widespread prelacteal feeding. Key determinants of EIBF were family type, receiving breastfeeding information, and partner support to breastfeed. Regarding prelacteal feeding, lower odds were observed among women with normal birth weight babies and those who received breastfeeding information. In contrast, a higher risk of prelacteal feeding was found among women experiencing delayed onset of lactation. Breastfeeding interventions and programs aimed at improving EIBF in the study area should focus on the women partners, nutrition counselling, and support to mothers from the extended family. To discourage prelacteal feeding practice, there is a need to intensify breastfeeding counselling and support to women with low birthweight babies and delayed onset of lactation.

## Supporting information

S1 Table Sociodemographic characteristics, breastfeeding practices and past obstetric history of the study sample. (DOCX)

S1 File English questionnaire. Questionnaire used for data collection.
(DOCX)
