# Supplementary material for: Prevalence and determinants of early initiation of breastfeeding (EIBF) and prelacteal feeding in Northern Ghana: A cross-sectional survey
Source: PLoS One. 2021 Nov 22;16(11):e0260347. doi: 10.1371/journal.pone.0260347 (PMC8608296; doi:10.1371/journal.pone.0260347)
Supplement: S1 Table — (DOCX) [file pone.0260347.s001.docx]

S1Table: Sociodemographic characteristics, breastfeeding practices and past obstetric history of the study sample

| Variable | Category | | Freq | % | Mean ±SD |
| --- | --- | --- | --- | --- | --- |
| Sociodemographic characteristics | | |  |  |  |
| Mother's Age (Years) |  | |  |  |  |
|  | < 30 | | 321 | 63.2 |  |
|  | ≥30 | | 187 | 36.8 | 27±5.2 |
|  |  |  |  |  |  |
| Marital status |  |  |  |  |  |
|  | Married | | 502 | 98.8 |  |
|  | Single | | 6 | 1.2 |  |
|  |  |  |  |  |  |
| Religion |  | |  |  |  |
|  | Islam | | 465 | 91.5 |  |
|  | Christianity | | 43 | 8.5 |  |
| Mother ethnic group |  | |  |  |  |
|  | Dagomba | | 424 | 83.5 |  |
|  | *Other ethnicities | | 84 | 16.5 |  |
| Level of education |  | |  |  |  |
|  | No formal education | | 269 | 53.0 |  |
|  | Primary | | 108 | 21.3 |  |
|  | Secondary | | 80 | 15.7 |  |
|  | Tertiary | | 51 | 10.0 |  |
| Type of family |  | |  |  |  |
|  | Nuclear family | | 176 | 34.6 |  |
|  | Extended family | | 332 | 65.4 |  |
| Working mother |  | |  |  |  |
|  | No  Yes | | 97  411 | 19.1  80.9 |  |
| Mother occupation |  | |  |  |  |
|  | Agric/farming | | 20 | 4.9 |  |
|  | Public/civil servant | | 44 | 10.7 |  |
|  | Trader | | 206 | 50.1 |  |
|  | Hairdresser/dressmaker | | 136 | 33.1 |  |
|  | Other occupation | | 5 | 1.2 |  |
| Mother place of work |  | |  |  |  |
|  | Home | | 87 | 21.2 |  |
|  | Outside home | | 324 | 78.8 |  |
| Wealth Quintile |  | |  |  |  |
|  | Poorest | | 106 | 20.1 |  |
|  | Second | | 96 | 19.0 |  |
|  | Middle | | 101 | 20.0 |  |
|  | Fourth | | 101 | 20.0 |  |
|  | Richest | | 101 | 20.0 |  |
| Number of Children |  | |  |  |  |
|  | <3 | | 255 | 50.2 |  |
|  | ≥3 | | 253 | 49.8 |  |
| Place of residence |  | |  |  |  |
|  | Rural | | 184 | 36.2 |  |
|  | Urban | | 324 | 63.8 |  |
| Age of infant (months) |  | |  |  |  |
|  | <10 | | 398 | 78.3 |  |
|  | ≥ 10 | | 110 | 21.7 | 6.5±5.2 |
| Child sex |  | |  |  |  |
|  | Male | | 243 | 47.8 |  |
|  | Female | | 265 | 52.2 |  |
| Normal Birthweight |  | |  |  |  |
|  | No | | 103 | 20.3 |  |
|  | Yes | | 405 | 79.7 |  |
| Early initiation of breastfeeding |  | |  |  |  |
|  | No | | 144 | 28.3 |  |
|  | Yes | | 364 | 71.7 |  |
| Pre-lacteal feeding |  | |  |  |  |
|  | No | | 404 | 79.5 |  |
|  | Yes | | 104 | 20.5 |  |
| Received breastfeeding information during pregnancy | | |  |  |  |
|  | No | | 103 | 20.3 |  |
|  | Yes | | 405 | 79.7 |  |
| Partner support to breastfeed | | |  |  |  |
|  | No | | 82 | 16.1 |  |
|  | Yes | | 426 | 83.9 |  |
| Delayed onset of lactation |  | |  |  |  |
|  | No | | 413 | 81.3 |  |
|  | Yes | | 95 | 18.7 |  |
| Previous breastfeeding experience |  | |  |  |  |
|  | No | | 114 | 22.4 |  |
|  | Yes | | 394 | 77.6 |  |
| Breastfeeding knowledge level | | |  |  |  |
|  | Low knowledge | | 38 | 7.5 |  |
|  | High knowledge | | 470 | 92.5 |  |
| Place of delivery |  | |  |  |  |
|  | Health facility | | 445 | 87.6 |  |
|  | Home | | 63 | 12.4 |  |
| Type of health facility |  | |  |  |  |
|  | Public health facility | | 416 | 93.5 |  |
|  | Private health facility | | 29 | 6.5 |  |
| Mode of delivery |  | |  |  |  |
|  | Vaginal delivery | | 454 | 89.4 |  |
|  | Cesarean delivery | | 54 | 10.6 |  |
| Birth attendant |  | |  |  |  |
|  | Skilled | | 444 | 87.4 |  |
|  | Unskilled | | 64 | 12.6 |  |
| Trimester of first ANC attendance |  | |  |  |  |
|  | First trimester | | 251 | 49.5 |  |
|  | Second trimester | | 250 | 49.3 |  |
|  | Third trimester | | 6 | 1.2 |  |
| Number of ANC visit |  | |  |  |  |
|  | Inadequate | | 318 | 62.6 |  |
|  | adequate | | 190 | 37.4 |  |
