# Supplementary material for: Prevalence and determinants of early initiation of breastfeeding (EIBF) and prelacteal feeding in Northern Ghana: A cross-sectional survey
Source: PLoS One. 2021 Nov 22;16(11):e0260347. doi: 10.1371/journal.pone.0260347 (PMC8608296; doi:10.1371/journal.pone.0260347)
Supplement: S1 File — Questionnaire used for data collection. (DOCX) [file pone.0260347.s002.docx]

**Study questionnaire:** Prevalence and determinants of early initiation of breastfeeding (EIBF) and prelacteal feeding in Northern Ghana: a cross-sectional survey

**Section A: sociodemographic information**

1. Age of mother in years ………………..
2. Marital status
3. Married
4. Single
5. Separated/divorced
6. Widowed
7. What religion do you belong to?
8. Islam
9. Christianity
10. Traditional
11. other specify ………….
12. What is your ethnic group?...........................
13. What is your level of education?
14. No formal education
15. basic education
16. Secondary
17. Tertiary
18. Do you live with other members of your family or you live with only your husband and children? (*If with only husband and children, tick nuclear otherwise, tick extended)*
19. Nuclear family
20. Extended family
21. Are you currently working?
22. Yes
23. No *If no, skip Q9*
24. If yes, what is your occupation?
25. Agric/farming
26. Public/civil servant
27. Trader
28. Hairdresser/dressmaker
29. Other specify……………
30. How many children do you currently have?.....................
31. Place of residence
32. Rural
33. Urban
34. Age of child ………………….months
35. Sex of child
36. Male
37. Female
38. Birth weight of child ( evident in MCH book)………………………………..

**Section B: Household wealth index**

1. Do you live in a rented or your own house?
2. Rented
3. Own house
4. What are the walls of your house you live in made of?
5. Sea sand/cement bricks
6. Cement/stone blocks
7. Mud and cement
8. Mud only
9. Are the walls of your house painted?
10. Yes
11. No
12. The floors of your room(s) is made of
13. Clay
14. Cement
15. Tiles
16. What is your house roof made of?
17. Aluminium/zinc sheets
18. Thatch/grass
19. What is your main source of drinking water in your household?
20. Tap water
21. Borehole
22. Rainwater
23. Stream/river water
24. What is your main source of light in the night in your household?
25. kerosene lamp
26. Candle
27. Electricity
28. Solar lamp
29. Torch light/battery powered
30. What is the main source of fuel for cooking in your household?
31. Charcoal
32. Firewood
33. Gas
34. Electricity
35. Does your household have any of the following?

*(circle the appropriate number in the table below (1. Yes, 0. No)*

|  | Yes | No |
| --- | --- | --- |
| Electricity | 1 | 0 |
| Television | 1 | 0 |
| Multi tv set | 1 | 0 |
| Washing machine | 1 | 0 |
| Computer/tablet | 1 | 0 |
| Video deck/DVD/VCD | 1 | 0 |
| Motorbike | 1 | 0 |
| Wall clock | 1 | 0 |
| Car | 1 | 0 |
| Bicycle | 1 | 0 |
| Landline telephone | 1 | 0 |
| Mobile phone | 1 | 0 |
| Radio | 1 | 0 |
| Freezer/refrigerator | 1 | 0 |
| Bed | 1 | 0 |
| Foam mattress | 1 | 0 |
| Sewing machine | 1 | 0 |
| Internet access | 1 | 0 |
| Table | 1 | 0 |

1. Do you own land?
2. Yes
3. No

**Section C: Breastfeeding and antenatal care attendance**

1. How long after birth did you first put (NAME) to the breast?

…...........minutes………….hours…………days

1. In the first three days after delivery, was (NAME) given anything to drink other than breast milk?
2. Yes
3. No
4. After birth, how many days did it take for your breastmilk to start flowing?

…………………….days

1. When you were pregnant for NAME, did you receive any information on breastfeeding?
2. Yes
3. No
4. Have you ever breastfed a child before NAME?
5. Where did you deliver NAME?
6. Health facility
7. Home
8. How was NAME delivered?
9. Vaginal delivery
10. Cesarean delivery
11. Who assisted you to deliver?
12. Doctor
13. Midwife/nurse
14. TBA
15. Others, specify…………..
16. I don’t know
17. Do you think your partner is supportive to you in breastfeeding (NAME)?
18. Yes
19. No
20. Did you ever attend ANC when you were pregnant for NAME? a. Yes
21. No, if NO, skip Q4.
22. If yes, indicate trimester of first ANC attendance? (*Enumerator should check from MCH and tick)*
23. First trimester
24. Second trimester
25. Third trimester
26. How many times did you attend ANC before giving birth to NAME? (*Enumerator should check from MCH book and tick)*

**SECTION D: Mothers level of Knowledge of breastfeeding**

1. For how long should breastfeeding start immediately after delivery?…………minutes …………..hours

***Preamble:*** *In this section, I will read a series of statements to you, you are expected to respond to each statement to indicate whether you agree or disagree by choosing* ***1= Yes , 0= No 3= Not sure***

1. Baby should be given something to eat before the start of breastfeeding. a. Yes
2. No
3. Not sure
4. The first milk that comes out of the breast after delivery should be discarded.
5. Yes
6. No
7. Not sure
8. Breast milk only is the best food for a newly born baby. a. Yes
9. No
10. Not sure
11. Newly born babies should not be given any food/water/juice apart from breast milk until the 6 month.
12. Yes
13. No
14. Not sure
15. Breast milk alone is enough for the baby for the first six months. a. Yes
16. No
17. Not sure
18. The baby should be breastfed anytime the baby wants breastmilk.
19. Yes
20. No
21. Not sure
22. Breastmilk does not protects babies from diseases.
23. Yes
24. No
25. Not sure
26. Exclusive breastfeeding helps to delay pregnancy.
27. Yes
28. No
29. Not sure
30. Breast milk contains all the nutrients the child needs up to the 6 month of life. a. Yes
31. No
32. Not sure
33. Breast milk is safe, hygienic and always available compared to infant formula. a. Yes

b No

b Not sure

1. Breastfeeding is beneficial to the mother, family, and the society. a. Yes

b No

c Not sure

1. Breastfeeding should be continued until the child is at least 24 months of age. a Yes

b No

c Not sure

1. A breastfeeding mother should eat a healthy diet to ensure the supply of breastmilk for the baby.
2. Yes
3. No
4. Not sure
